# Continuous 3D Printing of Biomimetic Beetle Mandible Structure with Long Bundles of Aramid Fiber Composites

**DOI:** 10.3390/biomimetics8030283

**Published:** 2023-07-01

**Authors:** Shuigen Li, Chang Liu, Yulong Zhang, Wei Zhang, Xuefei Xu, Zhaohua Lin, Yunhong Liang

**Affiliations:** 1 The Key Laboratory of Bionic Engineering, Ministry of Education, Jilin University, Changchun 130025, China; lisg21@mails.jlu.edu.cn (S.L.);; 2 Jilin Province Product Quality Supervision and Inspection Institute of Light Industrial and Chemical Products Inspection, Changchun 130022, China; 3 School of Mechanical and Aerospace Engineering, Jilin University, Changchun 130025, China; 4 Institute of Structured and Architected Materials, Liaoning Academy of Materials, Shenyang 110167, China

**Keywords:** self-made filament, bionic structure printing, continuous long bundle aramid fiber printing, 3D printing

## Abstract

Fiber-reinforced composites are an ideal high-performance composite material made from a combination of high-strength continuous fibers and a polymer matrix. Compared to short cut fibers, continuous long strand fibers can improve the mechanical properties of fiber composites more effectively. Herein, continuous aramid fiber-reinforced PLA filaments with fiber centering were prepared by modifying the outlet design of a desktop-grade thermoplastic single-screw melt extruder. Inspired by the cross-laminated structure of a beetle’s mandible fibers, a biomimetic structure composite was printed, which demonstrates a significant influence on the mechanical properties. The G-code printing program was developed, and the microstructure of the fracture surface of the specimen was analyzed. The uniform and orderly arrangement of aramid fibers within the PLA resin-based 3D-printed specimen was found. Consequentially, the bionic composites exhibits a 12% increase in tensile strength and a 5% increase in impact toughness, confirming the feasibility of utilizing continuous 3D printing to manufacture long bundles of aramid fiber composite filaments for enhanced mechanical performances.

## 1. Introduction

Fiber-reinforced polymers (FRPs) are composite materials that consist of high-strength continuous fibers, such as aramid fibers or carbon fibers, combined with a polymer matrix [1]. The fibers serve as the primary source of reinforcement strength, while the polymer matrix functions as a binder, safeguarding the fibers and facilitating the transfer of load. FRPs are generally regarded as high-performance polymer composites, renowned for their outstanding mechanical properties, particularly their remarkable specific strength and lightweight nature. In recent years, they have been utilized extensively in the aerospace, military, automotive, sports and prosthetics industries, catering to the demanding requirements of high mechanical performance [2,3,4,5,6]. Aramid fibers have gained significant attention as reinforcement materials in polymer composites due to their remarkable specific tensile strength, which is five times higher than that of steel wire, and their light weights, which are approximately 20% lighter than carbon fibers.

Compared to short fibers, continuous long-fiber bundles have the capability of significantly enhancing the mechanical performance of fiber composite materials [7,8]. Therefore, in the field of fiber-reinforced composites, the utilization of continuous long bundles of aramid fibers has emerged as a crucial focus of current research [9,10]. Polylactic acid (PLA) is a biodegradable polymer known for its excellent physical and mechanical properties [11]. It is one of the most extensively researched polymers in the field of thermoplastic fiber-reinforced composites [12]. Additive manufacturing technology, also known as 3D printing, is an emerging manufacturing process that builds solid parts by layering materials in a sequential manner [13,14]. It has gained significant utilization in diverse fields, including the aerospace, automotive and various other industries [15,16,17]. Fused deposition modeling (FDM) in 3D printing is characterized by its low cost and user-friendly features, making it the most widely utilized polymer additive manufacturing solution in fiber-reinforced composite materials [18]. Although FDM enables the forming of continuous fiber-reinforced composites, further research is needed to improve their mechanical properties. Bochnia, J., et al. studied the influence of different printing directions on the tensile mechanical properties of samples in FDM and found that there were significant differences in the mechanical properties of samples with different printing directions, which provided the idea for our research [19].

It has been reported that the beetle possesses a robust upper jaw, capable of withstanding substantial impacts, primarily attributed to the presence of a cross-laminated fiber structure on its surface. Compared to structures printed with parallel-oriented fibers, fiber-reinforced composite materials printed with special angle orientations exhibit higher impact toughness [20]. Inspired by the intersecting structure of beetles’ upper jaw fibers, a sample of a fiber composite material with an intersecting structure was prepared by 3D printing long bundles of fibers. In this study, self-built 3D printing equipment was utilized, and a self-made aramid fiber polylactic acid composite material was printed using a customized printing path program. The research successfully achieves the continuous printing of long bundles of aramid fiber composite materials and investigates the mechanical strength of 3D-printed fiber-reinforced composites. This study presents a novel and effective solution for printing continuous fiber composites with biomimetic structures, opening up new possibilities in the field of additive manufacturing.

## 2. Materials and Methods

### 2.1. Materials

Cyclommatus metallifer finae was purchased online (Black Cat’s Insect World Taobao store, Hangzhou, China). The 200D continuous aramid fiber composed of 200 Kevlar aramid fiber filaments was purchased from the DuPont Company of the United States. PLA particles (200,000, high molecular weight) were purchased from Nature Works in the United States, model number 4032D.

### 2.2. Preparation and Printing of Bionic Composites

#### 2.2.1. Preparation of Bionic Composite Filaments

**Figure 1:**
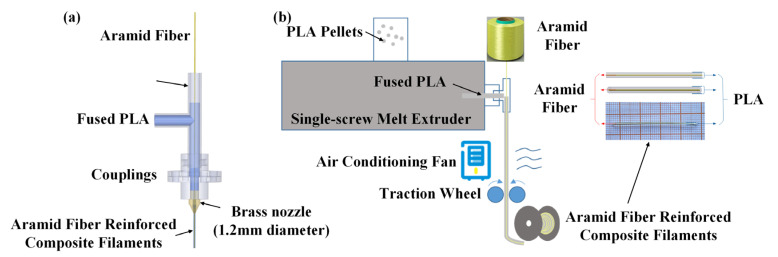
(**a**) Schematic diagram of the modification of the extruder, (**b**) schematic diagram the preparation of the aramid fiber-reinforced composite filament (The red line represents aramid fiber and the blue line represents PLA).

As shown in Figure 1, modifications were made to the extruder nozzle of the desktop FDM 3D printer filament single-screw melt extruder (Wellzoom, Shenzhen Mistar Technology Co., Ltd., Shenzhen, China) to enable the simultaneous extrusion of fibers and PLA, thereby fabricating 3D printing filaments with embedded fibers.

The PLA pellets were placed in a vacuum oven at 50 °C for 24 h to ensure proper drying. The dried PLA pellets were then poured into the feeding hopper of the 3D printer filament single-screw melt extruder. Through the process of melting and mixing, the PLA was extruded together with the aramid fibers at the nozzle, resulting in the production of continuous aramid fiber-reinforced composite filaments (with a filament diameter of 1.2 ± 0.1 mm).

#### 2.2.2. Printing of Bionic Composites

In order to achieve continuous printing of long bundles of aramid fiber-reinforced composite materials, traditional slicing software cannot be used to generate G-code codes. With the help of the tool developed by Andrew Gleadall—the FullControl G-Code Designer [21]—the printing path of each step was written. Finally, the G-Code file was previewed and modified using the slicing software Repetier-Host to enable the continuous printing of long fiber bundles.

**Figure 2:**
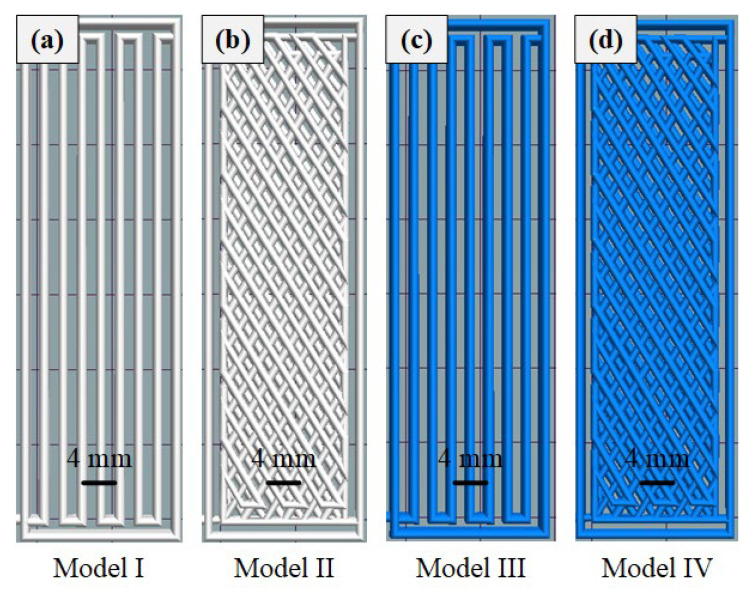
Schematic diagram of four printed structures (**a**) Model I (pure PLA without structure), (**b**) Model II (pure PLA bionic structure), (**c**) Model III (with fiber without structure) and (**d**) Model IV (with fiber bionic structure).

Four printed structures, as shown in Figure 2, named Model I (pure PLA without structure), Model II (pure PLA bionic structure), Model III (with fiber without structure) and Model IV (with fiber bionic structure), were designed to verify the effect of continuous fiber-reinforced composites and bionic beetle upper jaw structures on the strength of 3D-printed samples. The key technological parameters for 3D printing are shown in Table 1.

### 2.3. Characterization

#### 2.3.1. Structure Characterizations

To build a prototype of the biomimetic structure, the microstructure of the beetle’s upper jaw was analyzed with a scanning electron microscope (TESCAN VEGA4 tungsten lamp scanning electron microscope, Czech Republic). The distribution of long bundles of aramid fibers in the PLA matrix and the bonding interface between the aramid fibers and the PLA resin in Samples III and IV were also observed via scanning electron microscopy.

#### 2.3.2. Tensile Strength

A set of basic mechanical properties tests were carried out on self-made single aramid fiber composite filaments and pure PLA filaments to investigate the mechanical properties of the fiber reinforcement. Each filament had a diameter of 1.2 mm ± 0.1 mm and a length of 70 mm ± 0.2 mm. A C43 electronic universal testing machine (MTS Systems, Eden Prairie, MN, USA) was selected for the tensile test at a rate of 2 mm/min. A total of 10 test specimens were used for tensile test, and 5 specimens per filament for the same test.

To study the effect of the printed structures and the addition of long bundles of aramid fibers on the tensile mechanical properties of PLA specimens, the specimens were prepared with dimensions of 70 mm × 22 mm × 2 mm (length × width × height). The length and width errors of the four models printed in 3D were controlled to within ±0.3 mm, the height errors were controlled to within ±0.2 mm and the weight of the samples were controlled to within ±0.2 g. A total of 20 test specimens were used for tensile test, and 5 specimens per model for the same test.

Tensile experimental tests were carried out using an electronic universal testing machine (Model DDL100, Changchun Institute of Mechanical Science Co., Ltd., Changchun, China) with a constant loading rate of 2 mm/min.

#### 2.3.3. Impact Toughness

To study the effect of the printed structures and the addition of long bundles of aramid fibers on the impact mechanical properties of PLA specimens, the specimens were prepared with dimensions of 70 mm × 22 mm × 2 mm (length × width × height). The length and width errors of the four models printed in 3D were controlled to within ±0.3 mm, the height errors were controlled to within ±0.2 mm and the weight errors of the samples were controlled to within ±0.2 g. A total of 20 test specimens were used for impact testing, with 5 specimens per model used for the same test.

The impact toughness can be obtained from a JC-50D simple beam pendulum impact testing machine (Beijing Times Fenghua Technology Co., Ltd., Beijing, China). The loading span is 40 mm, using a 7.5 J pendulum with an impact speed of 3.8 m/s and an inclination angle of 160° for the toughness test. A total of 20 test specimens were used for the impact test, and 5 specimens per model were used for the same test.

## 3. Results and Discussion

### 3.1. Bionic Structure Design

**Figure 3:**
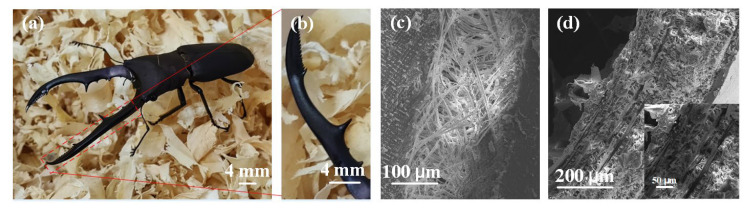
(**a**) Cyclommatus metallifer finae. (**b**) The mandible of Cyclommatus metallifer finae. (**c**,**d**) Microstructural features of the upper jaw of the Cyclommatus metallifer finae.

The mandible of the red stag beetle is highly regarded for its complex and beautiful structure, which is made up of a fibrous framework that plays a crucial role in protecting and supporting the beetle’s body. These fibrous structures are made up of a combination of chitin, proteins and other organic hardeners. The fibrous structure of the beetle’s palate may vary among different species and types of beetles to suit their respective environments and lifestyles. This complex fibrous structure makes the beetle’s upper jaw both rigid and flexible, providing adequate protection without restricting the beetle’s ability to move. The upper jaws of some beetles demonstrate a layered arrangement of fibers, creating an intricate interlaced fibrous network. This specific configuration significantly enhances the strength and toughness of the mandible, enabling it to withstand external impacts and pressures more effectively. These intriguing upper jaw structures of the beetle, as depicted in Figure 3, are excellent biological models for the design of biomimetic structures.

**Figure 4:**
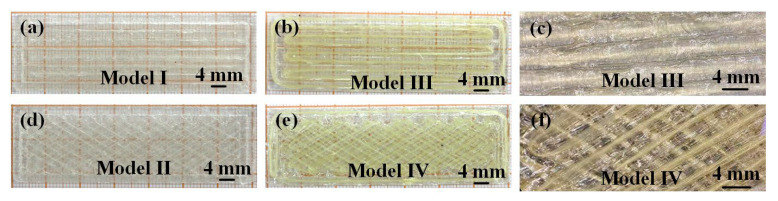
(**a**) A 3D-printed sample of Model I (pure PLA parallel structure), (**b**) a 3D-printed sample of Model III (aramid fiber-reinforced PLA parallel structure), (**c**) enlargement of Model III sample (observation through the OLYMPUS SZ61), (**d**) a 3D-printed sample of Model II (pure PLA bionic structure), (**e**) a 3D-printed sample of Model IV (aramid fiber-reinforced PLA bionic structure) and (**f**) enlargement of Model IV sample (observation through the OLYMPUS SZ61).

Four models (Model I, Model II, Model III and Model IV) were designed and analyzed to investigate the effect of long-bundle aramid fiber and biomimetic structure on the tensile and impact properties of PLA, as depicted in Figure 4. All models were designed with the same dimensions, measuring 70 mm × 22 mm × 2 mm (L × W × H).

**Figure 5:**
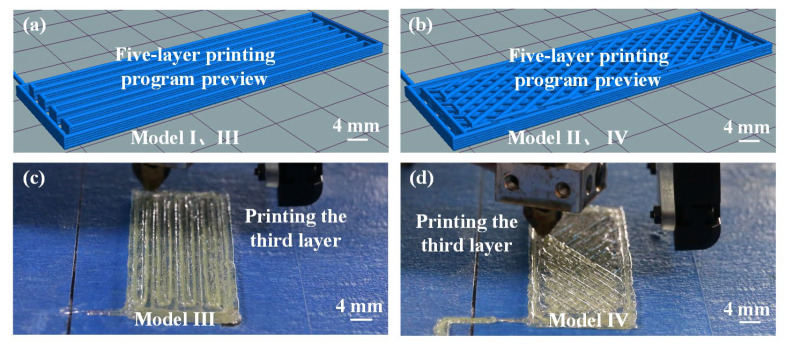
(**a**) The 3D-printed structures of Model I and Model III (five layers), (**b**) 3D-printed structures of Model II and Model IV (five layers), (**c**) the third layer of Model III being printed and (**d**) the third layer of Model IV being printed.

Figure 5a shows a preview of the printing procedure for Models I and III. Figure 5b shows a preview of the printing procedure for Models II and IV. Figure 5c,d show the third layer of aramid fiber composite being printed continuously on Models III and IV, respectively, where the fibers were uninterrupted from start to finish during the printing process. Models I and II were printed in pure PLA, without the use of aramid fibers, as shown in Figure 4a,d. Models III and IV, shown in Figure 4b,e, were printed by adding continuous aramid fibers to PLA resin. Figure 4d–f describe the design of the biomimetic mandible structure of Model II and Model IV, which consists of two intersecting fibers arranged at an angle of approximately 60° and repeated sequentially. Figure 4c,f show the fiber arrangements of Models III and IV, respectively.

The fill rate was 100%, and the mass fraction of aramid fibers was 5.2 wt% ± 0.3 wt%. The length and width errors of the four models printed in 3D were controlled to within ±0.3 mm, the height errors were controlled to within ±0.2 mm and the weight errors of the samples were controlled to within ±0.2 g. The structural data of the model are detailed in Table 2.

### 3.2. Mechanical Properties Analysis

**Figure 6:**
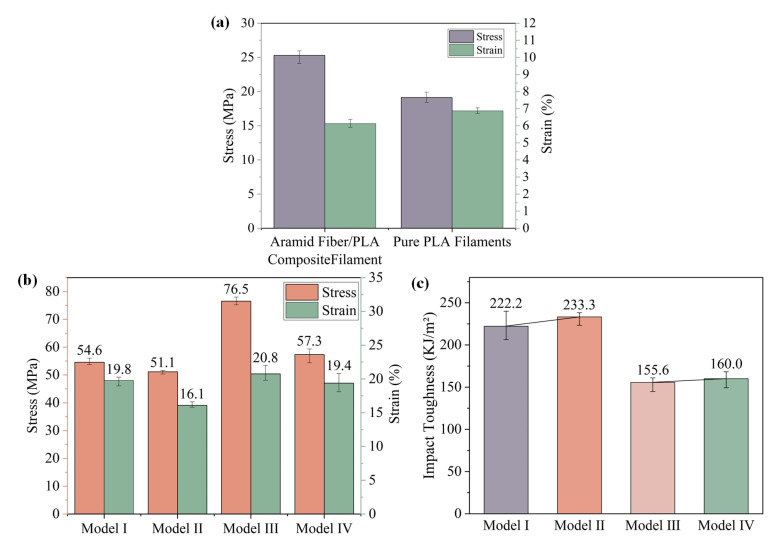
(**a**) Tensile mechanical properties of single aramid fiber/PLA and pure PLA filaments, (**b**) tensile mechanical properties of four models and (**c**) impact toughness of four models.

To assess the viability of the homemade aramid fiber-reinforced composite filaments, tensile tests were carried out on homemade aramid fiber/PLA and pure PLA filaments (Figure 6a). Compared to the tensile stress of 18.4–19.9 MPa for pure PLA filament, the tensile stress of the aramid fiber/PLA composite filament is 24.1–26 MPa, resulting in a 32% increase in tensile strength. This results demonstrated that self-manufactured long bundle aramid fiber can enhance the tensile performance of the composite filaments.

Moreover, to investigate the impact of biomimetic fiber crossing structures on mechanical strength, the mechanical properties such as tensile strength and impact toughness of biomimetic composites and biomimetic structural designs were investigated. Figure 6b shows the tensile properties of the four different models. The strain values for Models I, II, III and IV are 19–20.3%, 15.9–16.6%, 19.8–22% and 18.1–20.8%, respectively. The stress values of Models I, II, III and IV are 53.7–56.1 MPa, 50.3–51.7 MPa, 75.2–78 MPa and 54.4–59.4 MPa, respectively. When comparing the average stress values of four models with the same density, the tensile strengths of Model III and IV printed with aramid fibers and PLA composites were 40% and 12% higher, respectively, than those of Model I and II printed with pure PLA material. This comparison clearly shows that the incorporation of continuous long bundles of aramid fibers are able to increase the tensile strength of 3D-printed parts. It can be concluded that the aramid fiber-reinforced PLA composite achieves fiber reinforcement, thus demonstrating the feasibility of the fiber-reinforced composite filament preparation and the 3D printing method.

Figure 6c shows the impact toughness of the four various models. The impact toughness values for these models are 206.3–240 KJ/m^2^ (Model I), 221.2–241.6 KJ/m^2^ (Model II), 145.3–161.5 KJ/m^2^ (Model III) and 149.3–168.2 KJ/m^2^ (Model IV), respectively. Compared to the pure PLA used in Models I and II, the addition of aramid fibers in Model III and IV resulted in a 29.7% and 31.4% reduction in impact toughness, respectively. Conversely, the biomimetic structure in Model II and IV resulted in a 5% increase in impact toughness compared to the horizontally aligned Model I and III. These research findings demonstrate the notable advantages of bionic structural design in enhancing the mechanical performance of artificial materials [22].

Comparing Figure 6b,c, it can be seen that the tensile properties and impact properties of the 3D-printed biomimetic composites have been improved. Therefore, biomimetic composites are ideal for applications with large shock loads. These findings highlight the significance of material selection and structural design to achieve optimal mechanical properties for 3D-printed samples.

### 3.3. Microstructural Analysis

**Figure 7:**
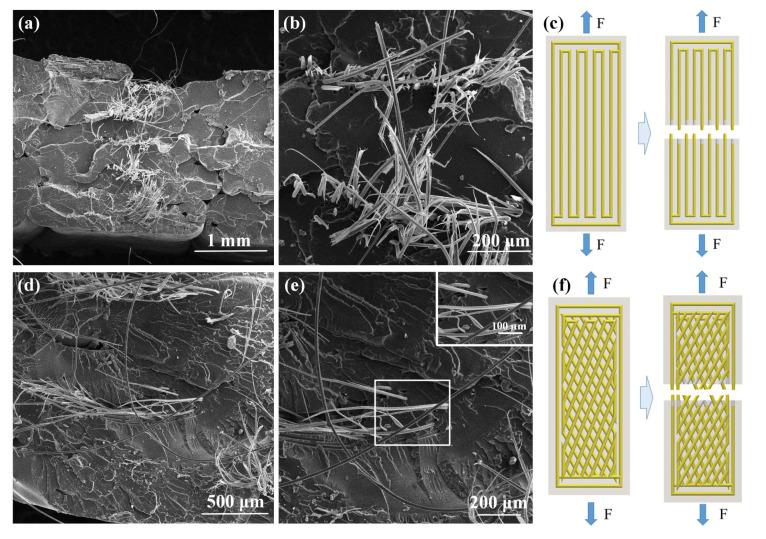
(**a**,**b**) SEM image of tensile fracture surface microstructure of Model III, (**c**) tensile test schematic of Model III, (**d**,**e**) SEM image of tensile fracture surface microstructure of Model IV and (**f**) tensile test schematic of Model IV.

To reveal the bionic fiber cross-structure and the effect of fiber reinforcement on the PLA reinforcement, the SEM of the tensile and impact fracture surfaces of Model III and IV was observed via electron microscope. Figure 7 shows the SEM images of the tensile fractured surfaces of Models III and IV and a schematic diagram of the tensile principle. The aramid fibers are encapsulated in PLA resin through 3D printing. Figure 4a,d shows that the aramid fibers are uniformly arranged within the print, indicating that the long bundles of aramid fiber-reinforced PLA composites were successfully printed. Specifically, Figure 4a,b depicts a parallel structure sample (Model III), in which the fractured portion of the matrix section is relatively flat. The bond between the aramid fibers and the PLA resin matrix is subjected to external stresses during the stretching process, causing the aramid fibers to break or pull out. Figure 4d,e shows the fractured microstructure of the bionic continuous aramid fiber 3D-printed composite (Model IV). Model IV has a slightly lower pull-up performance compared with the parallel structure (Model III) due to the fact that the long bundles of aramid fibers are not parallel to the tensile fracture surface and will be subjected to axial and radial loads during the stretching process.

**Figure 8:**
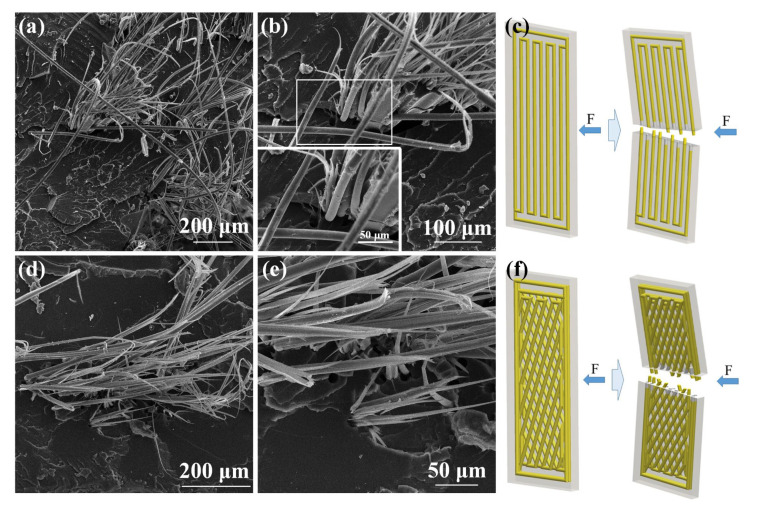
(**a**,**b**) SEM Image of impact fracture surface microstructure of Model III, (**c**) impact test schematic of Model III, (**d**,**e**) SEM image of impact fracture surface microstructure of Model IV and (**f**) impact test schematic of Model IV.

Figure 8 shows the SEM images of the impact fractured surfaces of Model III and IV and a schematic diagram of the impact principle. When the load impacts the composite material, the main damage mode of the high elastic modulus aramid fiber composite is fiber fracture and the crack propagation of the PLA matrix leads to the fracture of the sample, so the fracture propagation energy of the fiber composite is low. However, compared to Model III with the parallel structure, Model IV with the biomimetic structure displays better impact performance because the continuous aramid fibers at different angles can divide the impact force into axial force and radial force, preventing the crack from rapidly expanding in the PLA matrix. A bionic composite with a beetle’s mandible structure exhibits higher crack arrest performance compared to continuous aramid fibers aligned in parallel directions. The impact toughness of the bionic fiber composites is lower than that of pure PLA due to the weak interlayer bond between the fibers and the PLA.

Taken together, Figure 7 and Figure 8 demonstrate that continuous aramid fibers in biomimetic composites are critical to impeding crack propagation and absorbing impact forces. Crack extension can be prevented when a laminated fiber cross-web model IV sample is subjected to external forces. As a result, bionic composites with a beetle-like mandible structure exhibit superior crack arresting properties compared to parallel-aligned continuous aramid fibers. 

This study demonstrates the feasibility of 3D printing and emphasizes the significance of incorporating aramid fibers in the development of high performance composites. It provides a novel and effective fabrication method for 3D printing fiber-reinforced composites using PLA resin and continuous aramid fibers.

## 4. Conclusions

In this paper, a self-made filament based on aramid fiber-reinforced polylactic acid resin composite was utilized and a customized printing program was developed for 3D printing. The successful achievement of the continuous printing of aramid fiber composite material validated the feasibility of the self-made filament and the 3D printing method. Taking inspiration from the laminated fiber cross-web structure found in the upper jaw of a beetle, a bionic design resembling the structure of the beetle’s upper jaw has been meticulously crafted and brought to life through 3D printing technology. The fiber-reinforced composite bionic design with a specific angular structure improves mechanical properties, such as pull-up strength and impact toughness. 

During tensile fracture, the tensile strength of the fiber-reinforced composite is increased as the aramid fibers are encapsulated in the PLA resin and part of the load is consumed by the aramid fibers. The tensile strengths of the fiber-reinforced composites reached up to 78 MPa and 59.4 MPa, respectively, an increase of 40% and 12%, respectively compared to pure PLA. During impact fracture testing, the biomimetic composite material exhibited enhanced impact toughness due to the presence of a layered cross-web structure that hinders crack propagation. The lower impact toughness of fiber-reinforced composites compared to pure PLA is due to the weaker bond between the aramid fibers and the PLA resin. Therefore, the next step will be to investigate the bonding between the aramid fiber and PLA interface to achieve higher strength bionic structural composites for printing. The writing of the G code has a key influence on the quality of printed samples, as the G code controls the filling density of the samples, and only by writing the G code can we realize long bundle fiber printing. At the same time, the best mechanical properties of the printed samples can be achieved by adjusting various parameters in the G code. In the field of fiber-reinforced composite applications, the realization of self-made continuous long bundles of aramid fiber-reinforced composite filaments and continuous fiber printing represents a novel design and manufacturing method.

## Figures and Tables

**Table 1:** Key technological parameters for 3D printing.

	Model I	Model II	Model III	Model IV
Speed	120 mm/min	120 mm/min	120 mm/min	120 mm/min
Printing Rate	100%	100%	80%	75%
Layer Thickness	0.4 mm	0.4 mm	0.4 mm	0.4 mm
Nozzle Temperature	190 °C	190 °C	185 °C	185 °C
Nozzle Diameter	2 mm	2 mm	2 mm	2 mm
First Layer Bed Temperature	50 °C	50 °C	50 °C	50 °C
Remaining Layers Bed Temperature	50 °C	50 °C	45 °C	45 °C

**Table 2:** Printing parameters for four different models.

	Model I	Model II	Model III	Model IV
Materials	Pure PLA	Pure PLA	Aramid Fiber/PLA	Aramid Fiber/PLA
Structure	Paralleled	Bionic structures	Paralleled	Bionic structures
Printing speed	120 mm/min	120 mm/min	120 mm/min	120 mm/min
Length	70 ± 0.3 mm	70 ± 0.3 mm	70 ± 0.3 mm	70 ± 0.3 mm
Width	22 ± 0.3 mm	22 ± 0.3 mm	22 ± 0.3 mm	22 ± 0.3 mm
Height	2 ± 0.2 mm	2 ± 0.2 mm	2 ± 0.2 mm	2 ± 0.2 mm
Weight	4.2 ± 0.2 g	4.2 ± 0.2 g	4.2 ± 0.2 g	4.2 ± 0.2 g
Layer Height	0.4 mm	0.4 mm	0.4 mm	0.4 mm

## Data Availability

The data presented in this study are available upon request from the corresponding author.
